# A Treatise on Mechanical Dentistry

**Published:** 1843-06

**Authors:** Solyman Brown


### ARTICLE II.

### *A Treatise on Mechanical Dentistry.* By Solyman Brown, M. D., D. D. S.

(Concluded from page 202.)

### CHAPTER VI.

### *On Artificial Obturators and Palates.*

120. By artificial obturators are denoted contrivances for obstructing the passage of air, fluids or solid food, through the palatal arch, from the cavity of the mouth to the nasal openings, in cases where the bone and integuments of the arch are deficient by nature or removed by accident or disease. Artificial palates denote, as the words imply, complete substitutes for the natural palate, arched bone, or roof of the mouth, when that part is absent, from whatever cause.

121. Whether these deficiencies be congenital, as in the case of hare-lip, or the result of accident or disease, it is matter of little importance to the mechanical dentist to inquire, inasmuch as the preparation of the mouth, involving the medical and surgical treatment of the parts, does not belong to his department of the art. He should, however, be fully apprised that no mechanical contrivance of the nature of obturators and artificial palates should ever be applied to the mouth, until not only the natural teeth but the bony structure, muscular, membranous and other tissues are in a perfectly healthy condition.

122. Artificial palates are of two kinds, *simple* and complicated. They are *simple* when the object to be attained is merely to supply a deficiency in the palatal arch; and they are compound when, in connection with this main object, artificial teeth are required on the same plate. It sometimes happens that all the teeth have been removed from both jaws, in a mouth requiring an artificial palate; so that an entire double set is to be inserted in connection with it. This embraces the most comprehensive apparatus required at the hands of the mechanical dentist; and it may well be regarded as the most perfect test of his ingenuity and skill.

123. The evils to be remedied by artificial palates are of three kinds; first, inarticulate enunciation; second, the escape of food and fluids through the *nares;* third, difficulty of deglutition. Either of these evils, when any considerable portion of the natural parts is deficient, is of so grave a character, and constitutes so serious an embarrassment, that humanity requires the utmost efforts of the dental artist to remedy the defect; but when all these evils are felt by the same individual, the purest philanthropy will find itself not a little gratified when it can afford adequate relief to the unfortunate sufferer. It has rarely fallen to my lot to hear, from the mouth of any man, more ardent expressions of gratitude, than when restored to the power of proper articulation, such an individual speaks of the art and the artist that have restored him to himself and to society.

124. Attempts have been made to restore the *uvula* or hanging palate, when that organ has been removed; but the results have not warranted a belief that such an undertaking can ever be successful; at any rate in the present state of the arts and sciences. If the plate of the artificial palate be made to extend backward towards the *fauces* as far as possible, without irritating the surrounding tissues, the problem will be so far solved as to give the fullest satisfaction to every reasonable mind.

125. Although artificial obturators, sustained in the proper position by means of wings, sponge, or gum elastic, have been recommended by some dentists; among whom is the ingenious, industrious and liberal-minded Delabarre, the better opinion seems at present to prevail, that, in all cases where any contrivances must be used for the purposes before enumerated, pure gold and platina are the only substances which need to be employed; and that the metal should be sustained *in situ* by the same methods which are employed for supporting artificial teeth; viz. attachment to the natural teeth, atmospheric pressure, or spiral springs.

126. I shall, therefore, make no further allusion to any kind of obturators than such as I deem suited to the present improved state of dental practice, and these may be classed as *entire* or *partial artificial palates.* An artificial palate is *entire,* as the term denotes, when the whole of the palatal arch is covered by it; and it is *partial* when a small opening in the natural palate is covered by the metallic plate. These small openings, of which there are sometimes two or more in the same mouth, may occur in any part of the arch, and therefore require a peculiar construction of the artificial palates to suit this diversity of position.

127. It is believed that the well read and intelligent dentist who has perused the five preceding chapters of this treatise, and become familiar with the plates, will need no pictorial illustrations to enable him to understand this whole subject of artificial palates without any wood-cuts, or other visible representations of the methods and operations described. This fact, added to the reluctance which the author feels to subject the Journal to any further expense in connection with this treatise, especially in a time of unprecedented pecuniary depression in all sections of our country, and among all professions, will, he trusts, be his sufficient apology for using no plates in the present chapter.

128. If the defect in the natural palate consists of one small cavity through the bony arch, an exact model should be taken of the whole upper jaw embracing the large molars, to two or more of which the artificial palate may be attached by means of extensions or continuations of the principal plate, reaching to the teeth which the clasps are to embrace. The artificial palate should be thin and larger than the cavity, so that it may wholly cover it, and when thoroughly imbedded in its place, prevent the atmosphere from passing from the mouth to the nose and *vice versa*, through the opening. The clasps which sustain this plate may be so constructed as to be readily removed from the teeth, whenever the patient wishes to polish it and keep it clean. When the plate is small, two clasps, embracing healthy molar teeth, will be sufficient to support the plate; but when the orifice is large, it may be necessaay to attach clasps to three or more teeth. In case the orifice is very small and situated at the side of the arch near the teeth, a clasp may be so fitted as that a single tooth will sustain the apparatus. Sometimes it may be necessary to give a little elasticity to the plate or to parts of it, by the use of the planishing hammer, or by violent friction with a heavy burnisher. It is obvious that such a fixture, in case of small openings, will be less inconvenient than a larger plate extending quite across the mouth.

129. If, however, the teeth are closely set, and the dentist is reluctant to subject any of them to the action of clasps, let a thin gold plate be struck to a correct model of the palatal arch, extending quite to the teeth on every side, in the manner of a suction plate, and extending well backward towards the fauces. When we say that the plate must be struck so as to apply to all parts of the arch as a suction plate, we refer of course to those parts only of the arched roof of the mouth which remain in a natural state; for, where the parts are wanting, the models must be so fashioned, by the judgment of the artist, as to produce a plate of about the same shape and dimensions as would have been required to fit the palatal arch in its natural state, in order that the cavity of the mouth shall be of the same shape and capacity as though no deficiency had existed. If such a plate be well constructed, so as to press slightly against all the lateral and back teeth, and fit perfectly to all the fleshy parts which are in a natural state, it will remain in its place on the same principles as a common upper set held up by atmospheric pressure.

130. If, however, there be an opportunity to attach such a piece to one or more of the strong molars on each side of the mouth, it constitutes a regular artificial palate, of the most common and approved kind. But in case there be no good opportunity to secure an artificial palate, either by suction or to the teeth of the upper jaw, resort must be had to spiral springs, similar to those already described when treating of entire double sets of artificial teeth.

131. Before bringing this treatise to a conclusion, I feel myself in duty bound to urge upon my professional brethren the necessity and moral propriety of using their utmost efforts within the bounds of reason, to dissuade all their patients from the use of artificial teeth made of natural human teeth, sea-horse or other ivory, or any other substance liable to constant decomposition and decay. Gold, platina, and mineral teeth, should be the only materials used by the mechanical dentist, except in those rare instances where, in spite of his advice, some patient absolutely refuses to use these materials in consequence of prejudices which cannot be removed. Once, the first artists were compelled to use corruptible materials in the absence of better. But that day has passed, and a brighter era in dental practice has already arrived. Yet the full and immediate benefits of recent improvements will not be felt by the public at large, unless their importance shall be urged and explained by the members of our profession.

Natural human teeth and ivory have long enough held the place of better materials in dental practice, and since those better materials are now at hand, it is difficult to perceive any good reasons for perpetuating modes of practice, which are destined ere long to be known only in the history of the past.

132. In conclusion, the author tenders his grateful thanks to the great mass of his professional acquaintances, and to many strangers residing in remote sections of these states as well as beyond the ocean, who have expressed to him, by letter and otherwise, their approbation of the preceding chapters of this treatise. He takes this opportunity to say to them, that he has endeavoured to embrace in these chapters all the valuable information which it has been in his power to give, on the subject of which they treat. The liberality of this course has been censured indeed by a few individuals, who perhaps are honest if not generous in their opinion, that some facts should be kept secret in our profession, and used for personal emolument. The author, however, has thought otherwise, and having nothing to fear or to hope, from their censure or their praise, confidently submits the ultimate decision of the question to his brethren and their successors in the dental art.

